# Environmental T4-Family Bacteriophages Evolve to Escape Abortive Infection via Multiple Routes in a Bacterial Host Employing “Altruistic Suicide” through Type III Toxin-Antitoxin Systems

**DOI:** 10.3389/fmicb.2017.01006

**Published:** 2017-05-31

**Authors:** Bihe Chen, Chidiebere Akusobi, Xinzhe Fang, George P. C. Salmond

**Affiliations:** Department of Biochemistry, University of CambridgeCambridge, United Kingdom

**Keywords:** abortive infection, toxin-antitoxin, bacteriophage, *Serratia*, T4-family phage

## Abstract

Abortive infection is an anti-phage mechanism employed by a bacterium to initiate its own death upon phage infection. This reduces, or eliminates, production of viral progeny and protects clonal siblings in the bacterial population by an act akin to an “altruistic suicide.” Abortive infection can be mediated by a Type III toxin-antitoxin system called ToxIN_Pa_ consisting of an endoribonuclease toxin and RNA antitoxin. ToxIN_Pa_ is a heterohexameric quaternary complex in which pseudoknotted RNA inhibits the toxicity of the toxin until infection by certain phages causes destabilization of ToxIN_Pa_, leading to bacteriostasis and, eventually, lethality. However, it is still unknown why only certain phages are able to activate ToxIN_Pa_. To try to address this issue we first introduced ToxIN_Pa_ into the Gram-negative enterobacterium, *Serratia* sp. ATCC 39006 (*S* 39006) and then isolated new environmental *S* 39006 phages that were scored for activation of ToxIN_Pa_ and abortive infection capacity. We isolated three T4-like phages from a sewage treatment outflow point into the River Cam, each phage being isolated at least a year apart. These phages were susceptible to ToxIN_Pa_-mediated abortive infection but produced spontaneous “escape” mutants that were insensitive to ToxIN_Pa_. Analysis of these resistant mutants revealed three different routes of escaping ToxIN_Pa_, namely by mutating *asiA* (the product of which is a phage transcriptional co-activator); by mutating a conserved, yet functionally unknown, *orf84*; or by deleting a 6.5–10 kb region of the phage genome. Analysis of these evolved escape mutants may help uncover the nature of the corresponding phage product(s) involved in activation of ToxIN_Pa_.

## Introduction

Bacteria are susceptible to viral (bacteriophage) predation but have evolved several strategies to resist viral infection. One strategy is abortive infection (Abi) in which an infected bacterial cell dies precociously and thereby concomitantly blocks the production of mature phage progeny (Chopin et al., 2005). This protects clonal siblings in the bacterial population and therefore is akin to an “altruistic suicide.” Abi can be mediated through toxin-antitoxin (TA) systems, which are widespread in prokaryotes. Genetically, TA systems are usually composed of two genes transcribed from a single promoter. The upstream gene encodes an antitoxin that neutralizes the toxin product of the downstream gene. Functionally, TA systems impact on several biological processes such as the formation of persister cells, responses to environmental stress, plasmid stabilization, and phage exclusion through abortive infection (Gerdes et al., 2005).

Some Type III TA systems are bifunctional in that they also confer an Abi phenotype on their bacterial hosts. These TA systems are comprised of a proteinaceous toxin and an RNA antitoxin (Fineran et al., 2009). The first Type III TA system identified (ToxIN_Pa_) was encoded by a cryptic plasmid (pECA1039) in *Pectobacterium atrosepticum*. ToxIN_Pa_ was originally identified because ToxN shared 31% amino acid sequence identity with the AbiQ protein, which was involved in an abortive infection system in *Lactococcus lactis* (Emond et al., 1998; Fineran et al., 2009). Recent mutational analysis of the antitoxin and structural characterization of the toxin of the AbiQ system revealed that it is also a member of the Type III TA systems (Samson et al., 2013; Bélanger and Moineau, 2015). Since the discovery of ToxN_Pa_, numerous other Type III TA systems have been identified bioinformatically in diverse bacterial genera and they can be chromosomally, plasmid, or phage-encoded (Blower et al., 2012). Type III TA systems have been classified into ToxIN, CptIN, and TenpIN families. The toxic proteins of these families are homologous to ToxN_Pa_, while the antitoxin primary DNA sequences vary in length and number of tandem repeats. Interestingly, while ToxIN_Pa_ exhibited a strong Abi phenotype, ToxIN_Bt_ from *Bacillus thuringiensis* did not exhibit an Abi phenotype when challenged with over 100 different phages (Blower et al., 2012).

ToxIN_Pa_ is composed of an RNA antitoxin, ToxI, which binds to and suppresses the toxic endoribonuclease protein, ToxN. Crystallographic evidence revealed that ToxIN_Pa_ forms a triangular heterohexameric structure with 3 ToxN proteins in complex with 3 ToxI RNA pseudoknots (Blower et al., 2011). The complex is held in an inactive state under normal cellular conditions. However, during infection with specific phages, the complex is activated by an unknown mechanism, allowing the endoribonuclease to degrade host RNAs, leading to bacteriostasis and subsequent cell death. This phenomenon has features of a prokaryotic apoptosis that manifests itself in precocious death of the virally-infected bacterial host and, consequently, also inhibits progeny phage production. As this outcome restricts or terminates further phage invasion of the clonal bacterial population, the abortive infection process can be viewed as an altruistic suicide (Fineran et al., 2009).

Some phages aborted by ToxIN_Pa_ have the capacity to evolve spontaneous resistant mutants at low frequency that can circumvent the Abi system. The ToxIN_Pa_ resistant mutants of ΦTE, a *P. atrosepticum* phage, were found to overcome abortive infection by an RNA-based molecular mimicry of the ToxI antitoxin. The ΦTE escape phage had expanded a “pseudo-ToxI” region in the viral genome that was similar, but not identical, to the ToxI sequence. This expanded “pseudo-ToxI” region was expressed during phage infection and actively suppressed ToxN, thus allowing the ΦTE mutants to evade abortive infection (Blower et al., 2011). Recently, AbiQ-resistant mutations of four phages were sequenced revealing multiple loci involved in resistance, but how these genes conferred resistance to AbiQ remained elusive (Samson et al., 2013).

To date, ΦTE remains the only phage whose ToxIN_Pa_-escape mechanism is understood. Thus, the primary aim of this study was to characterize additional resistance mutations of new ToxIN_Pa_-sensitive phages and, by their study, perhaps add to the repertoire of known escape loci. Depending on the nature of the relevant mutation(s), the escape locus might provide insight into how phage infection leads to activation of the ToxIN_Pa_ complex.

In this study, we isolated and characterized *Serratia* sp. ATCC 39006 (*S* 39006)-specific phages, ΦCHI14, ΦX20, and ΦCBH8. Comparison of the genomic sequences of spontaneous ToxIN_Pa_-escape mutants and their wild type progenitors revealed three different routes of escape: (1) mutation of *asiA*, encoding a predicted phage transcriptional co-activator in ΦCHI14 and ΦCBH8; (2) mutation of an unknown gene (*orf84)* in a ΦCHI14 mutant; (3) deletion of a large region (6.5–10 kb) of the phage genome in most mutants of all three T4-family phages.

## Materials and methods

### Bacterial strains, bacteriophages, and growth conditions

Bacterial hosts and phages used in this study are listed in Table 1. All experiments were performed with *S* 39006. Bacteria were cultured at 30°C in Luria-Broth (LB) (10 g liter^−1^ tryptone, 5 g liter^−1^ yeast extract, 5 g liter^−1^ NaCl) or on LB agar (LBA) containing 1.5% w v^−1^ or 0.35% w v^−1^ agar to make LBA or top-LBA plates respectively. Bacterial growth was monitored by measuring optical density at 600 nm (OD_600_) using a Thermo Scientific Helios Zeta spectrophotometer. Where required, media were supplemented with ampicillin at 100 μg mL^−1^. Bacteriophages ΦCHI14, ΦX20, and ΦCBH8 were isolated from treated effluent collected from a sewage treatment plant in Cambridge, United Kingdom. The bacteriophages were selected from a library of *S* 39006-specific phages isolated using an enrichment procedure detailed previously (Evans et al., 2010). Phage lysates were generated as described previously (Petty et al., 2006). Spot tests were performed as described previously (Evans et al., 2010). Phages were stored at 4°C in phage buffer containing 10 mM MgSO_4_, 10 mM Tris-HCl, 0.01% w v^−1^ gelatin and a few drops of chloroform. Efficiency of Plating (E.O.P.) was calculated after incubating serial dilutions of phage lysates overnight on bacterial lawns on LBA and dividing the titer of the phage on the test host by the titer of the phage on the control host (Kutter, 2009).

**Table 1:** Bacterial strains, plasmids, and bacteriophages used in this study.

**Bacterial strain, plasmid, or phage**	**Relevant characteristics**	**References**
**STRAINS**		
*Serratia* sp. ATCC 39006 LacA (wt)	Laboratory strain, referred to as wild type (wt) in text, Lac^−^ derivative of *S* 39006, carbapenem+, prodigiosin+	
PLASMIDS^*^		
pTA46	*toxI*_Pa_, *ToxN_Pa_*	Fineran et al., 2009
pTA47	*toxI*_Pa_, *ToxN_Pa_-*frameshift (FS)	Fineran et al., 2009
pFR2	*tenpI_Pl_, tenpN_Pl_*	Blower et al., 2012
pFR8	*tenpI_Pl_, tenpN_Pl_*-FS	Blower et al., 2012
**BACTERIOPHAGES**		
ΦCHI14	Environmentally isolated phage	This study
ΦX20	Environmentally isolated phage	This study
ΦCBH8	Environmentally isolated phage	This study

* *All plasmids containing Type III TA loci are in a pBR322 vector with ampicillin resistance for selection. The TA complexes are expressed from their native promoter*.

### Isolation of phage escape mutants

Plaques of rare spontaneous phage mutants were isolated on lawns of *S* 39006 cells expressing ToxIN_Pa_ infected with wild type ΦCHI14, ΦX20, or ΦCBH8. Individual plaques were then purified at least twice on a lawn of *S* 39006 expressing ToxIN_Pa_. Final lysates of escape mutant phages were then prepared from near-confluent lawns until a final titer of >10^9^ plaque forming units (p.f.u.) mL^−1^ was obtained.

### Electron microscopy

Transmission Electron Micrograph (TEM) images of phages were taken at the Multi-Imaging Center, University of Cambridge using a Tecnai G2 series transmission electron microscope. Samples were prepared by adsorbing 10 μl of phage lysate (>10^8^ p.f.u. mL^−1^) onto a charged copper grid for 3 min. The grids were then washed with water twice before being stained with 2% phosphotungstic acid (PTA) neutralized with potassium hydroxide (KOH). The accelerating voltage was 120.0 kV and the direct magnification used to image phages was 25,000x.

### Phage genome sequencing

Phage DNA was extracted using a standard phenol-chloroform protocol (Sambrook, 1989). In a phase-lock gel (PLG) tube (5′ Prime), 450 μL of high titer phage lysate was incubated with 4.5 μL of 1 mg/mL DNase I and 2.5 μL of 10 mg/mL RNase A and incubated at 37°C for 30 min. The mixture was then added to 11.5 μL of 20% SDS and 4.5 μL of 10 mg/mL Proteinase K and incubated for another 30 min. DNA was extracted by adding 500 μL of a Phenol:Chloroform:Isoamyl Alcohol 25:24:1 mix and centrifuged at 1,500 × g for 5 min. The supernatant was transferred to a new PLG tube and the previous step repeated. In a new PLG tube, the supernatant was supplemented with 500 μL of Chloroform:Isoamyl Alcohol 24:1 and centrifuged at 1,500 × g for 5 min. The aqueous phase at the top was then incubated with 45 μL sodium acetate (3 mol/L, pH 5.2) and 500 μL of 100% Isopropanol at room temperature for 15 min. The mixture was then subjected to centrifugation at 12,000 × g for 20 min, after which the pellet was washed at least twice with 70% ethanol and then re-suspended in dH_2_O.

The genomes of wild type phages and selected escape mutants were sequenced using the Junior Roche 454 Genome Sequencer FLX pyrosequencer at the Department of Biochemistry, University of Cambridge or using the Illumina MiSeq, and HiSeq 2500 platforms at MicrobesNG. The resulting contigs were assembled using Genomic Sequencer *de novo* assembler (Roche) or SPAdes. The wild type sequences had coverage ranging from 57x to 100x of the full genome while the escape phage sequences had coverage ranging from 45x to 150x.

### Genome annotation and bioinformatics

Phage genome open reading frames (ORFs) were defined using the gene prediction tools GeneMark.hmm (Lukashin and Borodovsky, 1998) and Glimmer (Delcher et al., 1999). Homologs of predicted proteins were identified using PSI-BLASTp searches or i-TASSER (Roy et al., 2010). The program tRNAScan-SE (Lowe and Eddy, 1997) was used to identify phage tRNA genes and ARAGORN (Laslett and Canback, 2004) was used to predict host tRNA genes. The genomic sequences of wild type and escape phages were compared using Geneious 6.1 (Biomatters Ltd) and Artemis (Rutherford et al., 2000). Protein alignments were conducted using the EMBOSS “ClustalW” program. Final alignment images were generated using the ESPript 2.2 program. All the above analysis were used at default settings.

### DNA manipulations

The *asiA* locus of additional ΦCHI14 and ΦCBH8 mutants was probed by PCR amplification using primers oBH3 (5′- CTGTGACTTCGAGCTTAAATCTCC-3′) and oBH4 (5′- CGCTATATGTCAACAGGCCG-3′). Subsequent amplicons were subjected to Sanger sequencing.

### Phage burst size

Phage burst size assays were performed as described previously (Petty et al., 2007). In brief, an overnight *S* 39006 culture was used to inoculate LB in a 250 mL conical flask and incubated at 30°C to OD_600_ = 0.5. Phage samples were then added at a multiplicity of infection (M.O.I.) of 0.001 and the culture incubated with shaking at 150 rpm at 30°C. Samples were taken at different time points and chloroform-treated before titrating to determine the number of p.f.u. The one-step growth curve describes phages per initial infection center, over time.

### Phage adsorption

An overnight culture of *S* 39006 was adjusted to OD_600_ = 1 with LB in a 250 mL conical flask and infected with phages at an M.O.I. of 0.001. The 10 mL infected culture was placed in a shaking water bath at 30°C with shaking at 150 rpm. One hundred microliters samples were taken at different time points and added to 900 μL of chilled LB. The samples were chloroform-treated immediately and then titrated. The final adsorption curve was plotted by calculating the percentage of free phages in the culture against time. An LB-only sample was infected with phages as a negative control.

## Results

### Three ToxIN_Pa_-sensitive *S* 39006 phages were isolated

Since 2013, three phages that could be aborted by ToxIN_Pa_ were isolated from water from treated sewage effluent in samples taken at least a year apart from each other. The phages were initially isolated after enrichment on the *S* 39006 host expressing ToxIN_Pa_ with a frameshift mutation in the *toxN* gene. Abi sensitivity of these three phages, named ΦCHI14, ΦX20, and ΦCBH8 in chronological order, were examined initially by comparing titers from spot tests on *S* 39006 lawns expressing ToxIN_Pa_ or with the frameshifted version of the ToxIN_Pa_ locus as negative control. E.O.P. measurements showed that the three phages were strongly aborted by ToxIN_Pa_: the E.O.P.s of ΦCHI14, ΦX20, and ΦCBH8 were 2.0 × 10^−7^, 2.8 × 10^−8^, and 8.0 × 10^−6^ respectively. Therefore, all three phages could produce “spontaneous escape” mutants that became insensitive to ToxIN_Pa_ at low frequencies. Interestingly, all three phages were also aborted by another Type III TA system (TenpIN_Pl_) (Blower et al., 2012) but without producing any detectable spontaneous escape mutants (E.O.P. <10^−9^). None of the phages showed sensitivity to other Type III TA systems tested.

### ΦCHI14, ΦX20, and ΦCBH8 are T4-like phages of the myoviridae family

**Figure 1:**
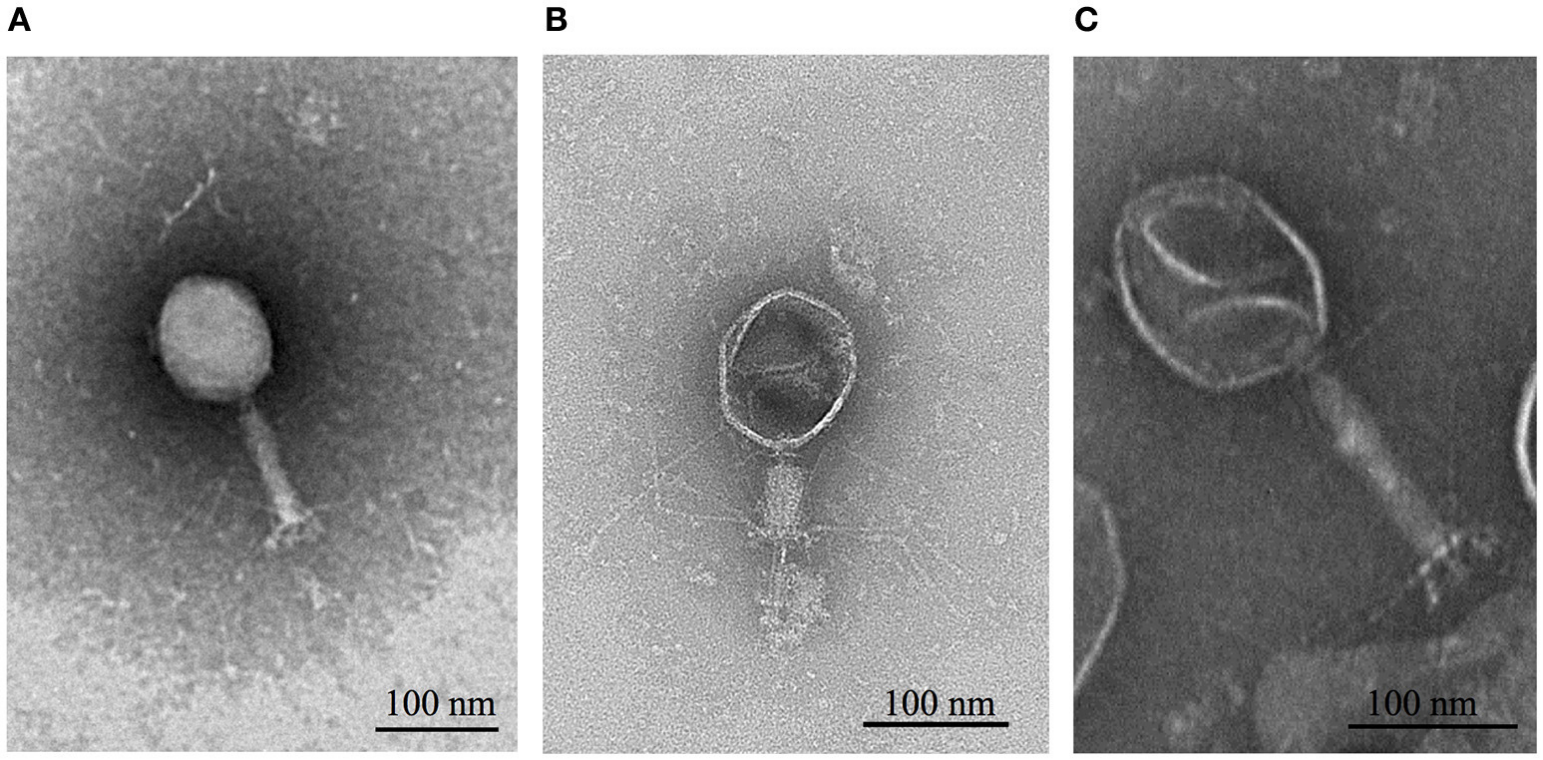
TEM images of **(A)** ΦCHI14, **(B)** ΦX20, and **(C)** ΦCBH8. ΦCHI14 and ΦCBH8 have extended tails while ΦX20 captured in the images shows a contracted tail.

TEM images revealed that all three phages had isometric, icosahedral heads, contractile tails and tail fibers (Figure 1). This classified them in the Caudovirales order and Myoviridae family (Ackermann, 2009). Whole genome sequencing results revealed that ΦCHI14, ΦX20, and ΦCBH8 were very similar to each other at the DNA sequence level. ΦCHI14 and ΦCBH8 both have a size of 171,151 bp and contain 275 predicted open reading frames (ORFs). ΦCHI14 and ΦCBH8 are almost identical genomically, except for 19 point mutations, 11 of which are in ORFs and cause non-synonymous mutations. tRNAscan-SE identified 16 tRNA genes encoded by ΦCHI14 and ΦCBH8. Similarly ΦX20 has a genome of 172,450 bp with 279 ORFs plus 17 predicted tRNA genes and it shared 93.8% homology with the genomes of ΦCHI14 and ΦCBH8. Interestingly all three phages encode almost twice the number of tRNAs as other related phages, such as T4 and CC31. Further, all three phages also have a GC content of ~38%, much lower than the host GC content of 49.24%. The genomes of all three phages were deposited in GenBank with the following accession numbers: ΦCHI14 (MF036690), ΦCBH8 (MF036691), and ΦX20 (MF036692).

**Figure 2:**
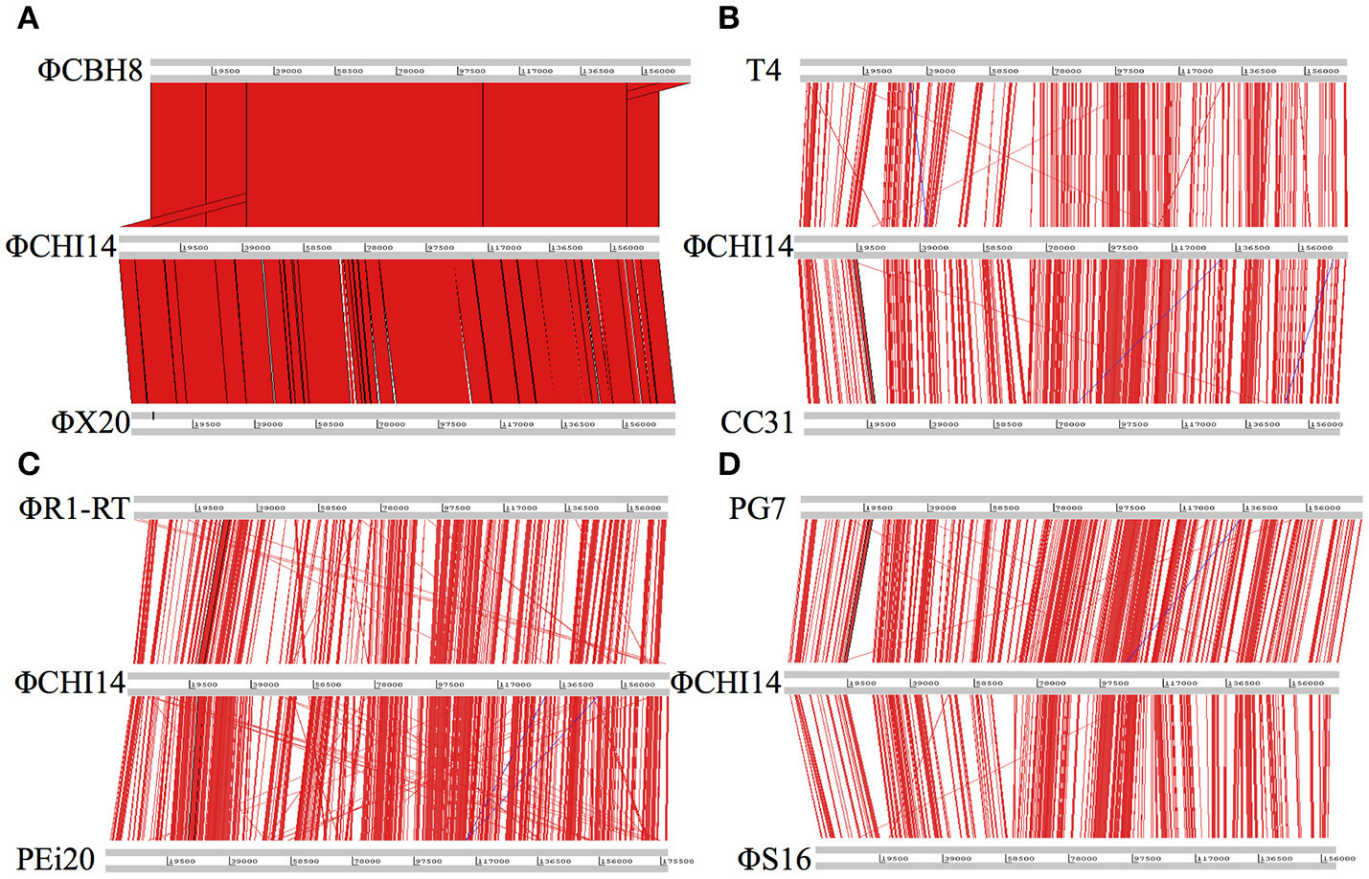
Whole genome alignment of wild type ΦCHI14 with other T4-like phages. DNA homologies are displayed in red. The degree of similarity is proportional to the intensity of red. Similar linear organization of the genome is observed between the aligned phages: **(A)** Comparison with ΦCBH8 and ΦX20. **(B)** Comparison with T4 and CC31. **(C)** Comparison with phage ΦR1-RT and PEi20. **(D)** Comparison with PG7 and ΦS16.

ΦCHI14 was used as a representative of the new isolates to compare with related phages (Figure 2): ΦCHI14 shares 55.0, 57.5, 55.3, 54.3, 57.4, and 60.1% DNA sequence identity with phages T4, CC31, ΦR1-RT, ΦS16, PG7, and PEi20 respectively—all of the above being T4-like phages.

### ToxIN_Pa_-sensitive *S* 39006 phages harbor at least three distinct ToxIN_Pa_ escape loci

Although ToxIN_Pa_ aborted T4-like *S 39006* phages efficiently, rare phage plaques appeared at low frequencies. We presumed that these rare plaques arose due to viral mutations enabling phages to “escape” or circumvent the effects of ToxIN_Pa_. Therefore, we expected that definition of the corresponding mutations would enable identification of the phage genes encoding products responsible for “activation” of the ToxIN_Pa_ system.

In total, 24 “escape” mutants of these *S* 39006 phages were subject to whole genome sequencing; 5 of ΦCHI14 mutants, 11 of ΦX20 mutants and 8 of ΦCBH8 mutants. Mutations were mapped to the corresponding wild type genomes to identify the putative ToxIN_Pa_ resistance loci. Interestingly, the mutations did not all map to single locations in the corresponding genomes. Instead, three distinct mutational types were identified in the genomes of the 24 escape phages (Table 2): 21 mutants harbored a large deletion (size ranging from 6.5 to 10 kb) in their genomes; 2 mutants carried a nonsense mutation or deletion in the *asiA* gene; and 1 mutant had a missense mutation in an *orf* encoding a hypothetical protein, ORF84.

**Table 2:** Summary of mutations in ΦCHI14, ΦX20, and ΦCBH8 ToxIN_Pa_ escape mutants.

**Escape phage**	**Mutation type**	**Gene(s) affected**	**Effect of mutation**
ΦCHI14a	large deletion (7,647 bp)	14 ORFs	elimination of affected genes
		12 tRNA genes	
ΦCHI14b	nonsense mutation	*asiA*	E71 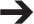 stop codon
ΦCHI14c	large deletion (10,094 bp)	19 ORFs	elimination of affected genes
		13 tRNA genes	
ΦCHI14e	missense mutation	*orf84*	E66D
ΦCHI14f	deletion (10 bp)	*asiA*	extends protein by 17 residues
ΦCBH8f	large deletion (10,040 bp)	19 ORFs	elimination of affected genes
		11 tRNA genes	
ΦCBH8l	large deletion (7,802 bp)	15 ORFs	elimination of affected genes
		11 tRNA genes	
ΦCBH8m	large deletion (8,575 bp)	17 ORFs	elimination of affected genes
		6 tRNA genes	
ΦCBH8o	large deletion (6,521 bp)	14 ORFs	elimination of affected genes
		10 tRNA genes	
ΦCBH8p	large deletion (8,368 bp)	16 ORFs	elimination of affected genes
		10 tRNA genes	
ΦCBH8t	large deletion (7,328 bp)	15 ORFs	elimination of affected genes
		9 tRNA genes	
ΦCBH8u	large deletion (8,158 bp)	17 ORFs	elimination of affected genes
		4 tRNA genes	
ΦCBH8x	large deletion (7,731 bp)	14 ORFs	elimination of affected genes
		10 tRNA genes	
ΦX20b	large deletion (9,533 bp)	19 ORFs	elimination of affected genes
		11 tRNA genes	
ΦX20d	large deletion (9,479 bp)	19 ORFs	elimination of affected genes
		8 tRNA genes	
ΦX20f	large deletion (9,473 bp)	19 ORFs	elimination of affected genes
		8 tRNA genes	
ΦX20g	large deletion (9,533 bp)	19 ORFs	elimination of affected genes
		11 tRNA genes	
ΦX20h	large deletion (9533 bp)	19 ORFs	elimination of affected genes
		11 tRNA genes	
ΦX20j	large deletion (9,533 bp)	19 ORFs	elimination of affected genes
		11 tRNA genes	
ΦX20k	large deletion (9,533 bp)	19 ORFs	elimination of affected genes
		11 tRNA genes	
ΦX20l	large deletion (9,533 bp)	19 ORFs	elimination of affected genes
		11 tRNA genes	
ΦX20m	large deletion (9,533 bp)	19 ORFs	elimination of affected genes
		11 tRNA genes	
ΦX20n	large deletion (9,533 bp)	19 ORFs	elimination of affected genes
		11 tRNA genes	
ΦX20o	large deletion (9,533 bp)	19 ORFs	elimination of affected genes
		11 tRNA genes	

### Asia is involved in the activation of ToxIN_Pa_

**Figure 3:**
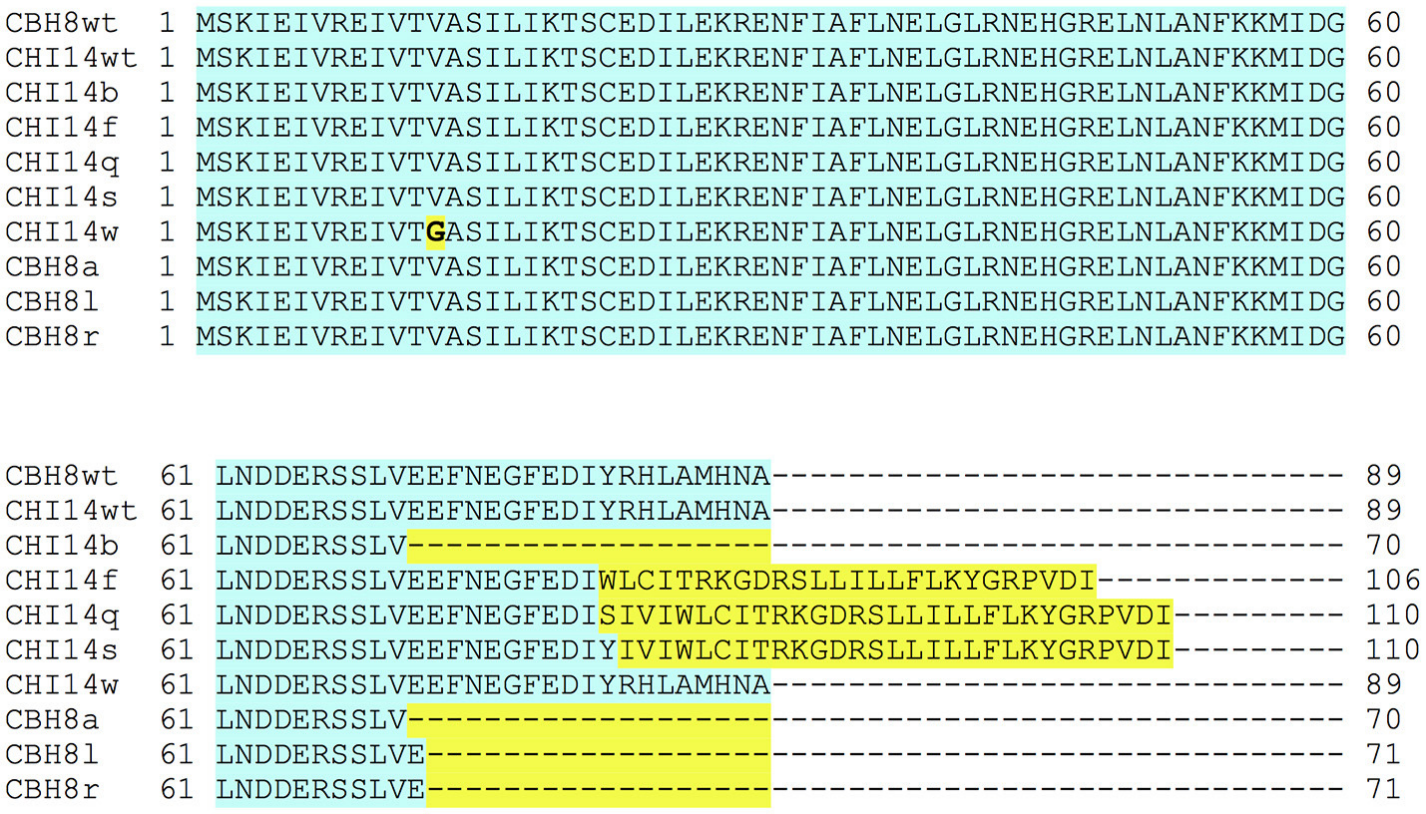
Alignment of the primary sequence of AsiA encoded in ΦCHI14 and ΦCBH8 wild type and escape phages. Blue highlight indicates identical sequences while yellow highlight shows variations in the primary sequence of AsiA in escape mutants.

Two sequenced escape mutants of ΦCHI14 (ΦCHI14b and ΦCHI14f) had mutations in the *asiA* gene (Table 2). After defining these mutant *asiA* variants, the *asiA* locus in additional ΦCHI14 and ΦCBH8 “escape” mutants were probed by PCR and Sanger sequencing and 6 further independent *asiA* mutants were identified. The *asiA* locus encodes the protein AsiA, the homolog of which in ΦT4 is involved in σ^70^-appropriation (Hinton et al., 2005). Most of the mutations affect the C-terminal domain (CTD) of AsiA, including: (1) truncation of the CTD via a point mutation leading to a premature stop codon; (2) extension of the CTD via insertion or deletion of nucleotides leading to frameshift mutations that eliminated the natural stop codon. Only one mutant had a V13G mutation in the N-terminal domain of the protein (Figure 3).

### ORF84 may be involved in the activation of ToxIN_Pa_

One mutant (ΦCHI14e) had a single nucleotide substitution in the gene, *orf84*. The A to C substitution caused an E66D change in the encoded ORF84 protein. Homologs of ORF84 have been found in 11 other phages, the most similar homolog belonging to enterobacterial phage, CC31. However, no functional information exists currently for any of the homologs from both sequenced-based predictions and structure-based predictions.

### The “large deletion” is the most prevalent mutational route through which these *S* 39006 phages escape ToxIN_Pa_

**Figure 4:**
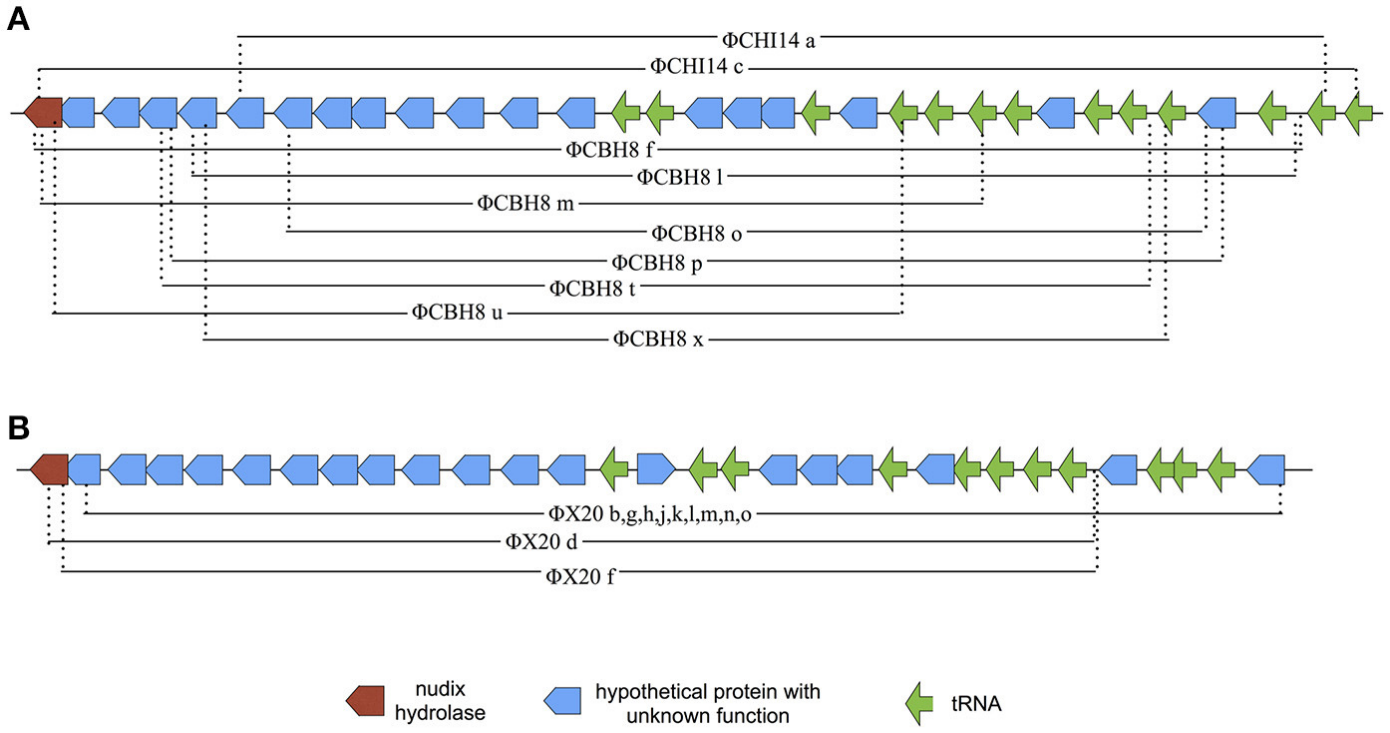
Diagram of the deleted regions in ΦCHI14, ΦCBH8, and ΦX20 mutants. **(A)** Mapping of “large deletion” regions onto ΦCHI14 and ΦCBH8 genomes shows the start and end of each deletion. The same presentations of the deleted regions are used for ΦCHI14 and ΦCBH8 due to their high similarity. **(B)** Mapping of “large deletion” regions onto the ΦX20 genome shows the start and end of each deletion.

The majority (21 out of 24) of the sequenced “escape” mutants of *S* 39006 phages became insensitive to ToxIN_Pa_ by a similar type of mutation—through a large deletion of a specific viral genome locus (Table 2). This was the only common mutational route through which all three *S* 39006 phages could escape ToxIN_Pa_. In particular, all 11 of the ΦX20 mutants isolated arose through this “large deletion” mutation. The corresponding deletions overlapped across a common core and varied from 6,521 bp (ΦCBH8o) to 10,094 bp (ΦCHI14c). The largest deleted region contains 19 ORFs and 13 tRNA genes, whereas the smallest contains 13 ORFs and 10 tRNA genes (Figure 4). Although the precise 5′ and 3′ borders of most deletion mutations were variable, closer inspection revealed the presence of direct repeats flanking the deleted region in every mutant (Table 3). The repeat length and sequence was unique in each mutant and most of the repeats appeared numerous times in the wild type genome, some are also represented within the deleted region. The presence of the direct repeats may suggest slipped mispairing during replication, or inter/intra-molecular misalignment during recombination, as possible mechanisms driving the deletion mutations (Singer and Westlye, 1988; Pierce and Masker, 1989).

**Table 3:** Sequences of direct repeat flanking the “large deletion” region in mutants are shown.

**Mutant name**	**Repeat sequence**	**Frequency of repeat in wild type genome**	**Frequency of repeat within deleted region**
ΦCHI14a	TCAGCCA	40	3
ΦCHI14c	GGATTA	>100	4
ΦCBH8f	GAACTGC	22	3
ΦCBH8l	TTGAGTAG	7	0
ΦCBH8m	GTCCCTG	11	0
ΦCBH8o	CCGAAGC	15	1
ΦCBH8p	GTTCAC	94	4
ΦCBH8t	AGCCATCC	5	0
ΦCBH8u	GGAAGCC	22	1
ΦCBH8x	ATCTG	>100	11
ΦX20b, g, h, j, k, l, m, n, o	AACTGCTACA	2	0
ΦX20d	GGGAAAC	6	0
ΦX20f	CTTCGCC	21	1

*The repeat length varies from 5 to 10 nucleotides and the sequences are different in each mutant of unique deletion size. Most of the repeats appear numerous times in the wild type genome and some of them appear also within the deleted region*.

#### Deleted regions contain mostly unknown ORFs

The ability of “large deletion” mutants to escape ToxIN_Pa_ infers that the 6.5–10 kb deleted region contains genetic elements directly or indirectly responsible for activating ToxIN_Pa_. In our limited pool of spontaneous “large deletion” mutants, 6.5 kb was the most compact deletion and therefore detailed inspection of the ORFs and tRNAs in this region was undertaken. Every individual predicted ORF was investigated by searching for DNA homologies using nucleotide BLAST; amino acid sequence homology using protein BLAST; and finally i-TASSER for predicted structural homologs.

Of the 13 predicted ORFs within the smallest deletion region found in ΦCBH8o, 6 encode hypothetical proteins with no sequence, or structural, homologs, while 7 of the ORFs encode homologs in other T4-like phages. However, no functional information is available about any of these homologs—except for one hypothetical protein encoded by the 3′ end of the deletion region. This hypothetical protein, (ORF145), shows some similarity to the membrane anchor domain of an agglutinating adhesin (YadA) in both protein BLAST and i-TASSER structural predictions.

**Figure 5:**
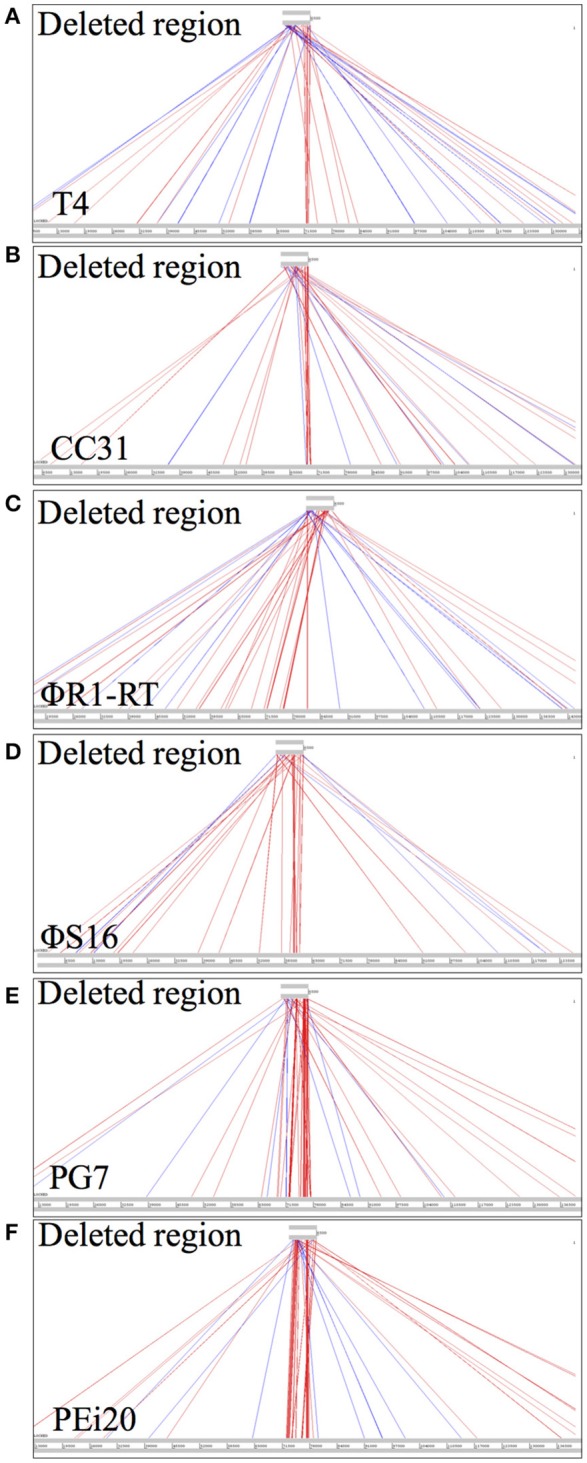
DNA sequence comparisons of the smallest deleted region in ΦCBH8o (6.5 kb) with other T4-like phages, using ACT. **(A)** Comparison with phage T4. **(B)** Comparison with CC31. **(C)** Comparison with ΦR1-RT. **(D)** Comparison with ΦS16. **(E)** Comparison with PG7. **(F)** Comparison with PEi20.

The absence of any functional information for individual ORFs in the “large deletion” locus prompted an analysis of whether the region is present in other related phages or is unique to the T4-family *S* 39006 phages reported in this study. Therefore, the 6.5 kb smallest deletion region from ΦCBH8o was aligned with the genomes of the related phages that show high homology with the entire genomes of these environmental T4-like *S* 39006 phages. ACT alignment showed only very limited identity of the 6.5 kb region with phages T4, CC31, ΦR1-RT, ΦS16, PG7, and PEi20 (Figure 5).

The presence of multiple tRNA genes within the deleted regions may also suggest that these tRNAs play a role in susceptibility to ToxIN_Pa_. Among all the “large deletion” mutants, ΦCBH8u showed deletion of the smallest number of tRNAs: Gly with the TTC anticodon, Met with the CAT anticodon, Arg with the TCT anticodon and Leu with the TAA anticodon. tRNAs genes with the same anticodons for the above mentioned amino acids appear 3 times and 6 times for the first two and only once for the last two in the host genome. The presence of these tRNA genes in the host genome suggests it may be unlikely that they are the direct activators of ToxIN_Pa_. However, the low frequency of Arg (TCT) and Leu (TAA) in the host genome suggests the possibility that their deletion from the phage genome might lead to inefficient translation of some viral transcripts encoding products that activate ToxIN_Pa_.

#### Loss of the large deletion locus affects the fitness of mutant phages

**Figure 6:**
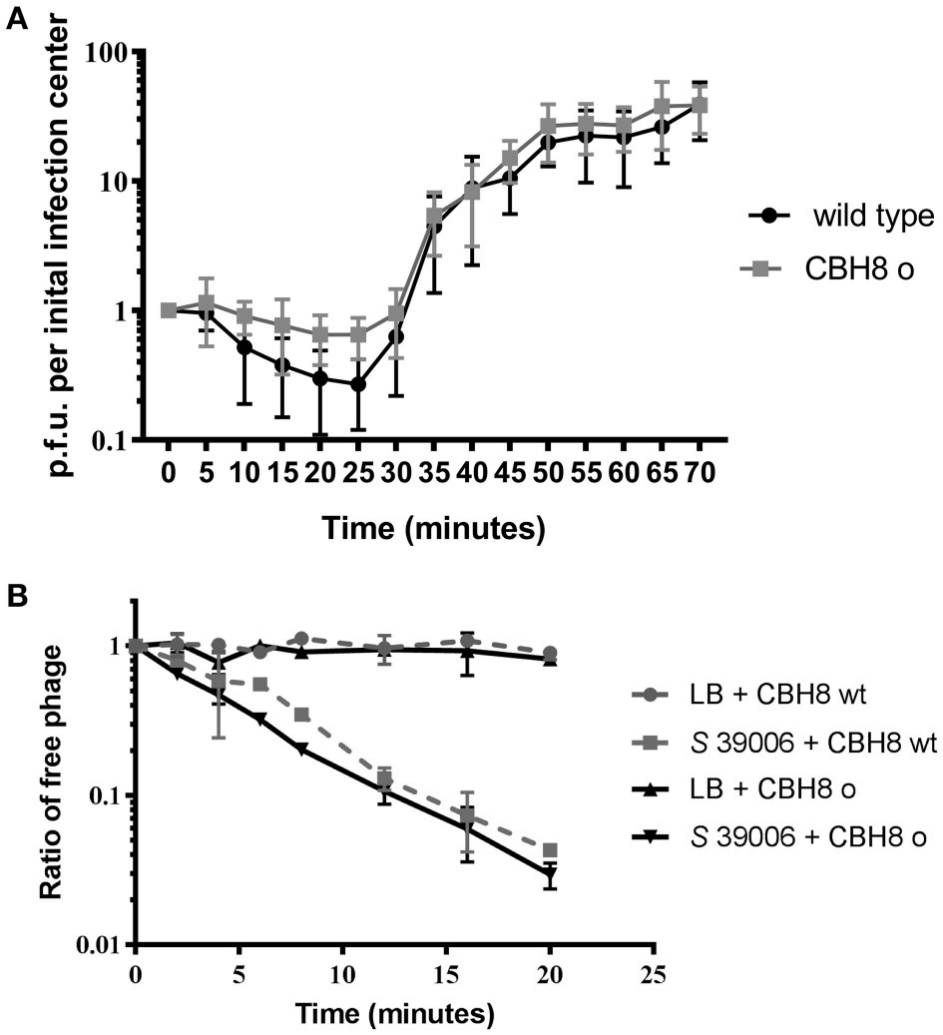
Fitness comparison of ΦCBH8wt and ΦCBH8o. **(A)** One-step growth curve of ΦCBH8wt and ΦCBH8o. **(B)** Adsorption of ΦCBH8wt and ΦCBH8o to wild type *S* 39006. ΦCBH8wt and ΦCBH8o with LB-only was used as a negative control for adsorption.

Given the extent of the viral genome deletions and absence of any functional information on most of the deleted gene products, we decided to investigate the impact of the deletions on phage fitness, represented by adsorption efficiency and burst size. The burst size of wild type ΦCBH8 and ΦCBH8o infecting exponential phase *S* 39006 with an M.O.I. of 0.001 was measured by one-step growth (*n* = 5). Both wild type ΦCBH8 and ΦCBH8o showed a latent period of 25 min and a rise period of about 35 min. The average burst size was approximately 22 phage particles per initial infection center for wild type ΦCBH8 and 28 for ΦCBH8o (Figure 6A). However, the adsorption efficiency of ΦCBH8o was lower than that of wild type ΦCBH8 (Figure 6A), suggesting a fitness defect. To investigate if the difference in adsorption was phage-dependent or host-dependent, we carried out a second adsorption assay (*n* = 3) using stationary phase *S* 39006 instead of exponential phase host but retaining the same M.O.I. As shown in Figure 6B, no obvious difference in binding to host cells was observed—both phages achieving >95% adsorption in 20 min. These results may suggest that the “large deletion” mutation causes decrease in adsorption efficiency only when infecting exponential phase host cultures, but causes no significant difference in burst size. However, due to the variable nature of the fitness assays, further experiments should be carried out in the future to verify the differences in adsorption seen in ΦCBH8 and the ΦCBH8o mutant, especially in the exponential phase of bacterial growth.

## Discussion

In this study we isolated three highly homologous T4-family environmental phages of *S* 39006 that are sensitive to ToxIN_Pa_-mediated Abi. Rare spontaneous mutants of these phages were able to circumvent ToxIN_Pa_ via multiple escape routes. The independent isolation of ΦCHI14, ΦX20, and ΦCBH8 is interesting since pairwise alignment of their genomes with other T4-like phages showed that they are mosaics of one another—whole-genome alignments consist of varying-length stretches of high homology interspersed with stretches of no homology (Nolan et al., 2006; Petrov et al., 2006). This kind of similarity and diversity is typical among T4-like phages (Petrov et al., 2010) yet the relatively low overall sequence similarities indicate that ΦCHI14, ΦX20, and ΦCBH8 may represent a new type of T4-like genome configuration. Furthermore, the isolation of only 3 ToxIN_Pa_-sensitive, and very similar phages (despite multiple environmental enrichments yielding more than 300 phages over a 3-year span) suggests a relatively rare incidence of ToxIN_Pa_-sensitive viruses among environmental phages of *S* 39006. However, this rarity may not be surprising because wild type *S* 39006 does not carry ToxIN_Pa_ naturally. Furthermore, it is possible that these new phages arose in the environment by propagation on alternative native hosts taxonomically unrelated to *S* 39006.

We showed recently that a single phage gene product from phage ΦM1 was responsible for activating the ToxIN_Pa_ system in the natural host, *P. atrosepticum* (Blower et al., 2017). A phage product might interact with ToxI and degrade it or sequester it away from ToxN to liberate the toxin. Alternatively, a phage product could interact with ToxN, reducing the affinity between ToxN and ToxI. Interactions of the phage product(s) with both ToxI and ToxN are also formally possible. Based on our recent observations with the evolution of ΦM1 to ToxIN_Pa_ resistance in *P. atrosepticum*, we expected to find mutations in a single locus in “escape” mutants of ΦCHI14, ΦX20, and ΦCBH8. However, our characterization of the latter “escape” mutants has now suggested a more complicated landscape for potential activation mechanisms operating on ToxIN_Pa_.

**Figure 7:**
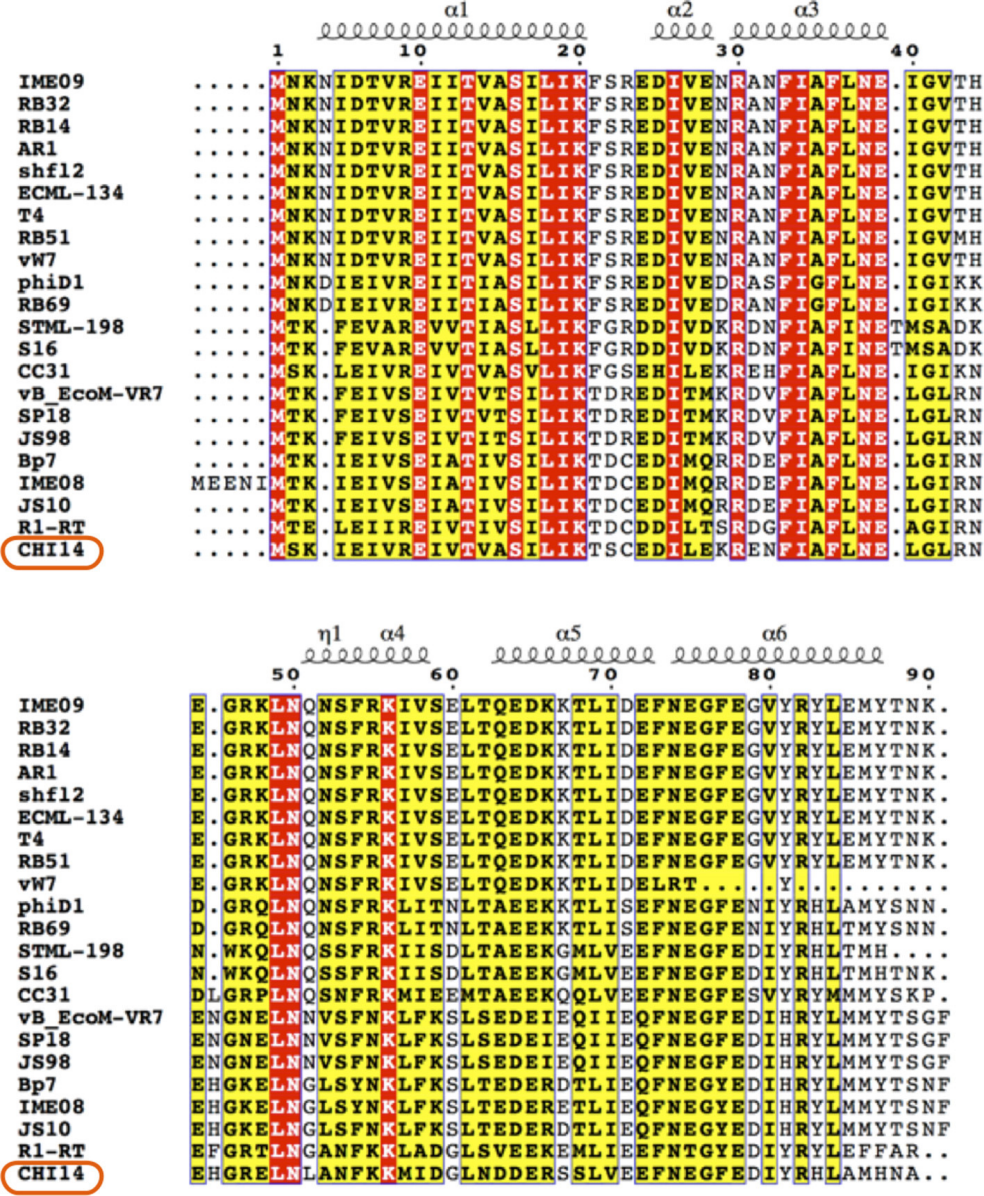
Alignment of AsiA from ΦCHI14 and related phages. The N-terminal region of AsiA is highly conserved among phages while the C-terminal region is more varied. The protein secondary structure prediction is overlaid on the alignment.

One “escape” locus found in both ΦCHI14 and ΦCBH8 mutants is *asiA*, which encodes AsiA, a highly-conserved protein in T4-like phages (Figure 7). In T4, AsiA is an anti-sigma factor that represses host transcription through a process known as σ-appropriation, and, together with MotA, co-activates transcription of phage middle genes (Hinton et al., 2005). AsiA monomers bind tightly to regions 4.1 and 4.2 of σ^70^ and abolish the sigma factor's ability to bind to the −35 promoter sequence of host genes (Hinton, 2010). As a result, AsiA inhibits transcription of bacterial genes with a −10/−35 promoter. In addition, AsiA-bound σ^70^ adopts an altered conformation that allows the T4 transcriptional activator, MotA, to bind σ^70^ and the MotA box present in the promoter region of T4 middle-expressed genes. Thus, the AsiA-σ^70^-MotA complex disrupts the ability of RNA-polymerase (RNAP) to recognize and transcribe host genes but reconfigures the enzyme to transcribe phage middle-expressed genes (Ouhammouch et al., 1995; Lambert et al., 2004). Most of the 6 escape mutants of ΦCHI14 and ΦCBH8 produce an AsiA with either an extended or truncated CTD, while only one mutant has a V13G substitution in the NTD. In AsiA the role of the CTD is controversial and not fully understood, with some studies showing that mutations in the CTD of AsiA affected its function in σ-appropriation and MotA binding (Yuan and Hochschild, 2009; Yuan et al., 2009) while other studies showed no compromise in σ-appropriation of AsiA with a mutated CTD (Pal et al., 2003). As for the V13G mutation in the NTD, the mutated amino acid is homologous to V14 in T4, which was defined as an essential residue for AsiA-AsiA homodimer and AsiA-σ^70^ interactions (Gilmore et al., 2010). Future research will investigate whether AsiA plays a direct role in ToxIN_Pa_ activation as a phage product or as a part of the AsiA-RNAP-MotA complex, or whether AsiA indirectly activates ToxIN_Pa_ by disturbing ToxI:ToxN stoichiometry or phage middle gene transcription.

The most frequently found escape locus among the *S* 39006 phage mutants was the deletion of a substantial region of the phage genomes. The size of the deletion represented 3.8–5.8% of the total genome size. These deletant mutants remained viable on *S* 39006 indicating that the deleted region is not essential for phage replication, at least under the laboratory conditions used. How the deletion mutations confer resistance is not immediately obvious, given the number of genes affected. As no missense mutations were isolated that mapped to this locus, we presume that two or more genes have to be mutated in this locus simultaneously for phages to escape, and presumably, these genes might be located toward the two ends of the deletion, hence the large deletion size. An interesting hypothesis here would be that the entire deletion region might contain genes that wild type phages acquired by recombinogenic lateral gene transfer (Petrov et al., 2010) in the natural environment (perhaps during mixed viral infections) and these genes, in concert, could be responsible for activating ToxIN_Pa_ in bacterial hosts. The possibility that the deleted region was acquired through lateral gene transfer is supported by two pieces of evidence: (1) attempts to align the large deletion locus with several other T4-like phages (T4, CC31, ΦR1-RT, ΦS16, PG7, and PEi20) showed very little homology (Figure 5); and (2) most of the 5' borders of the large deletion mutations lie within tRNA genes (Figure 4), whose conserved sequences are readily recombinogenic. It could be argued that this has echoes of the descriptions of Pathogenicity Islands in bacteria (Karaolis et al., 1999; Schmidt and Hensel, 2004) and therefore could even suggest that the large deletion region is a potentially mobile viral genome unit.

The only clue to possible functions of ORFs in the deleted regions comes from ORF145, which is a YadA homolog. YadA was first found in *Yersinia* species and serves as a virulence factor that mediates *Yersinia* adherence to epithelial tissue (Hoiczyk et al., 2000). YadA cognate genes have also been found in other phages but it is unknown if they might be transferred to their bacterial hosts to enhance virulence (Moreno Switt et al., 2013). Future work will involve investigating the impact of ORF145 in allowing phage “escape.” Finally, given the important physiological roles for phage-encoded tRNAs in viral morphogenesis, future research into the role that the tRNAs encoded within the deleted regions may play in phage “escape” requires investigation.

In summary, this study has discovered a distinct group of T4-family *S* 39006 phages that could activate the ToxIN_Pa_-mediated abortive infection system. The data on the “escape” mutants of these environmental phages suggest that either ToxIN_Pa_ can be activated by more than one phage product, or that production of the ToxIN_Pa_- activating product involves multiple biological processes and that defects in several could enable phages to circumvent Abi. Future investigations will involve further characterization of the products from the escape loci of these phages, and experiments to try to dissect their physiological roles during the process of abortive infection—and its circumvention.

## Author contributions

GS, BC, CA, and XF conceived and designed the experiments. BC, CA, and XF performed the experiments. BC and CA prepared figures and graphs. BC and GS wrote the manuscript. All the authors read and approved the final manuscript.

### Conflict of interest statement

The authors declare that the research was conducted in the absence of any commercial or financial relationships that could be construed as a potential conflict of interest.
